# Supplementary material for: Suspected Pituitary Apoplexy: Clinical Presentation, Diagnostic Imaging Findings and Outcome in 19 Dogs
Source: Vet Sci. 2022 Apr 15;9(4):191. doi: 10.3390/vetsci9040191 (PMC9026492; doi:10.3390/vetsci9040191)
Supplement: Supplementary file 1 [file vetsci-09-00191-s001.zip › vetsci-1616409-supplementary.pdf]

Table S1. Detailed description of neurological examination findings for each dog. Pupillary light reflex (PLR). No alterations detected (\).

Signalment	Behaviour	Mental status	Posture	Gait	Postural reactions	Cranial nerves evaluation	Epileptic seizures	Hyperalgesia
1. Mixed breed MN, 9.75 y, 24.6 Kg	\	\	\	\	\	\	No	\
2. Italian hound F, 11.5 y, 14.8 Kg	\	Obtundation	\	\	\	\	No	\
3. mixed breed FN, 12.7 y, 4.7 Kg	\	Obtundation	\	Proprioceptive ataxia hindlimbs	Deficit left anterior and posterior limb	\	No	Cervical
4. Italian bracco dog F, 12.8 y, 27 Kg	Compulsion, circling, head pressing	Obtundation	Neck vetroflexion	Hindlimbs hypometria	Deficit 4 limbs	\	No	Cervical
5. Mixed breed FN, 12.7 y, 30 Kg	Vocalizations	\	\	\	\	\	No	\
6. Beagle FN, 7.6 y, 16.6 Kg	\	\	\	\	\	\	No	\
7. Labrador retriever F, 5 y, 31.2 Kg	Anxious behaviour	\	\	\	\	\	Yes	\

8. Labrador retriever FN, 10.8 y, 35 Kg	\	Obtundation	\	\	\	\	Yes	\
9. Mixed breed FN, 5.8 y, 21 Kg	Compulsive behaviour	\	\	\	Deficit 4 limbs	Reduced menace response right side	No	Diffuse
10. Labrador retriever FN, 8.6 y, 29 Kg	\	\	\	\	\	\	Yes	\
11. Boxer FN, 11.4 y, 27.4 Kg	Disorientation, circling, compulsive behaviour	\	\	\	\	\	No	\
12. Springer Spaniel M, 13.7 y, 18.7 Kg	Disorientation, anxious behaviour	\	\	\	\	Bilaterally absent menace response, bilaterally absent direct and consensual PLR	Yes	\
13. Mixed breed M, 11.1 y, 33 Kg	Compulsive behaviour, circling	\	\	\	Deficit left hindlimb	Bilaterally absent menace response	No	\
14. Mixed breed M, 9.8 y, 14.5 Kg	Disorientation	\	\	\	\	\	No	\

15. Corso dog FN, 9.4 y, 43 Kg	\	\	\	Tetraparesis	\	\	No	\
16. English bulldog M, 6.1 y, 28 Kg	Disorientation, head pressing, aggressive behaviour	Obtundation	\	\	\	Bilaterally absent menace response, bilaterally reduced direct and consensual PLR	No	\
17. Mixed breed M, 9.3 y, 9.3 Kg	\	Obtundation	\	\	\	\	Yes	\
18. Mixed breed M, 3.2 y, 15 Kg	Disorientation, aggressive behaviour	\	\	Proprioceptive ataxia 4 limbs	Deficit 4 limbs	\	Yes	\
19. Mixed breed M, 11.1 y, 27.6 Kg	Disorientation, vocalizations	Obtundation	\	\	\	\	No	\
